# Temporal Changes in Cell Marker Expression and Cellular Infiltration in a Controlled Cortical Impact Model in Adult Male C57BL/6 Mice

**DOI:** 10.1371/journal.pone.0041892

**Published:** 2012-07-24

**Authors:** Xuemei Jin, Hiroshi Ishii, Zhongbin Bai, Takahide Itokazu, Toshihide Yamashita

**Affiliations:** 1 Department of Molecular Neuroscience, Graduate School of Medicine, Osaka University, Suita-shi, Osaka, Japan; 2 Core Research for Evolutional Science and Technology (CREST), Japan Science and Technology Agency (JST), Chiyoda-ku, Tokyo, Japan; 3 Key Laboratory of Puer Tea Science, Ministry of Education, Yunnan Agriculture University, Kunming, China; University of Lyon, France

## Abstract

**Background:**

Traumatic injury to the central nervous system (CNS) triggers a robust inflammatory response that leads to axonal damage and secondary degeneration of spared tissue. In contrast, some immune responses have neuroprotective effects. However, detailed information regarding the dynamics of immune responses after traumatic CNS injury is still unavailable.

**Methods:**

In the present study, changes in the immune cells present in the injured brain, spleen, and cervical lymph nodes (CLNs), which are draining lymphatic organs from the CNS, were analyzed after controlled cortical impact (CCI) by flow cytometry and immunohistochemistry.

**Results:**

The number of neutrophils and macrophages that infiltrated the injured brain immediately increased 1 d post-injury and declined rapidly thereafter. In the injured brain, resident microglia showed a bimodal increase during the first week and in the chronic phase (≥3 weeks) after injury. Increase in the Iba-1^+^ microglia/macrophages was observed around the injured site. Morphologic analysis showed that Iba-1^+^ cells were round at 1 week, whereas those at 3 weeks were more ramified. Furthermore, CD86^+^/CD11b^+^ M1-like microglia increased at 4 weeks after CCI, whereas CD206^+^/CD11b^+^ M2-like microglia increased at 1 week. These results suggest that different subsets of microglia increased in the acute and chronic phases after CCI. Dendritic cells and T cells increased transiently within 1 week in the injured brain. In the CLNs and the spleen, T cells showed dynamic changes after CCI. In particular, the alteration in the number of T cells in the CLNs showed a similar pattern, with a 1-week delay, to that of microglia in the injured brain.

**Conclusion:**

The data from this study provide useful information on the dynamics of immune cells in CNS injuries.

## Introduction

The central nervous system (CNS) is anatomically separated from the rest of the body and has been considered an immunologically privileged site [1], [2]. The important anatomical features of the CNS include the following: (a) lack of lymphatic drainage from the parenchyma; (b) lack of endogenous antigen-presenting cells (APCs); and (c) the blood-brain barrier (BBB) or blood-spinal cord barrier (BSCB), which restricts the access of soluble factors to the CNS and limits the access of immune cells to the site [3]–[5]. However, immune cells such as neutrophils, macrophages (bone marrow-derived macrophages), T cells, and dendritic cells (DCs) may infiltrate brain parenchyma after injury to the CNS, by penetrating breaks in the BBB or BSCB [6]–[8]. Once immune cells have infiltrated the CNS, they may release reactive oxygen species, nitrogen oxide, free radicals, and proteases, which can exacerbate tissue damage [9]–[11]. Leukocytes that have infiltrated the CNS also release cytokines and chemokines, which activate the resident microglia or blood-derived monocytes to participate in the immune response at the injured sites [12], [13]. In contrast, activated microglia and macrophages play both beneficial and harmful roles in the injured CNS [14]–[17]. Under inflammatory conditions, extrinsic cells such as neutrophils, macrophages, T cells, and DCs interact with resident microglia to maintain equilibrium between the injured CNS and the immune system [18]–[20].

**Figure 1:**
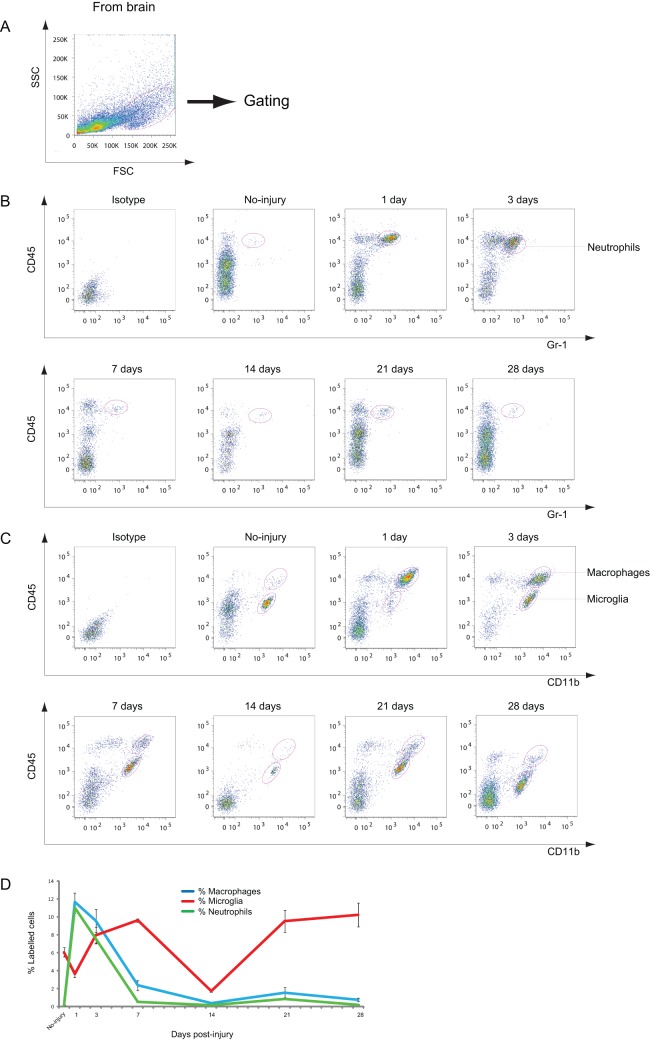
Analysis of neutrophils, macrophages, and microglia in the injured brain. A: Dot plots of isolated immune cells in the brain, gated for live cell analysis. B: Representative cytometry data for neutrophils (CD45^+^/Gr-1^+^ cells) in the injured brain at the indicated days after CCI. C: Representative cytometry data for macrophages (CD45^high^/CD11b^+^ cells) and microglia (CD45^low^/CD11b^+^ cells) in the injured brain at the indicated days after CCI. D: Graph illustrating quantitative data for accumulated neutrophils, macrophages, and microglia in the injured brain up to 28 d after CCI. All the values are presented in terms of mean ± standard error of mean (*n* = 3–6). Macrophages/neutrophils: ** *p*<0.01 at 1, 3, and 14 dpi compared with no injury; microglia: ** *p*<0.01 at 14 and 28 dpi compared with no injury, * *p*<0.05 at 14 dpi compared with 1 dpi.

**Figure 2:**
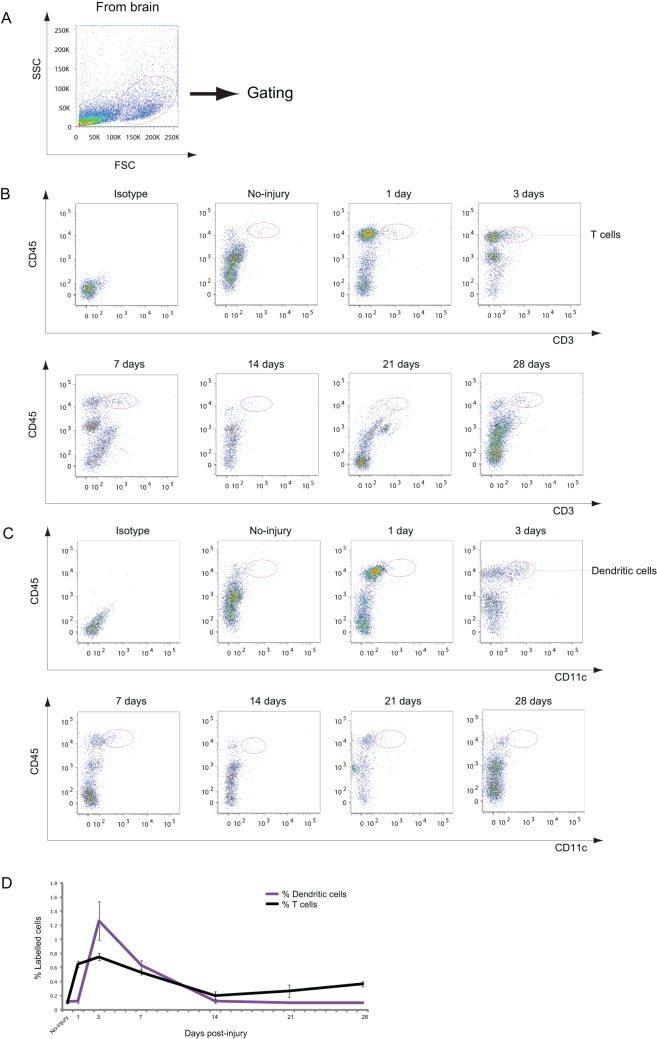
Analysis of T cells and dendritic cells in the injured brain. A: Dot plots of isolated immune cells in the brain, gated for live cell analysis. B: Representative cytometry data for T cells (CD45^+^/CD3^+^ cells) in the injured brain at the indicated days after CCI. C: Representative cytometry data for CD45^+^/CD11c^+^ dendritic cells (DCs) in the injured brain after CCI. D: Graph illustrating quantitative data for accumulated T cells and DCs in the injured brain after CCI. *n* = 3–6 for each experiment. T cells: ** *p*<0.01 at 1, 3, and 7 dpi compared with no injury, * *p*<0.05 at 28 dpi compared with no injury; DCs: ** *p*<0.01 at 3 and 7 dpi compared with no injury.

T cells are considered harmful to the injured CNS after traumatic brain injury (TBI) [21], [22]. However, T cells may also have neuroprotective effects, which contribute to repair [23]. Under an inflammatory milieu in the CNS, APCs interact with meningeal T cells, which home to cervical lymph nodes (CLNs) via lymphatic vessels [24]. Several studies have shown that antigen-carrying DCs participate in restricting damage to the nervous system after trauma to the CNS and during the process of post-injury repair [25]. DCs emigrating from the brain have been shown to infiltrate peripheral lymphatic organs, inducing a local immune response and directing antigen-specific T cells back to the brain [26]–[28]. Notably, in rodents and ruminants, the cerebrospinal fluid (CSF) flows into the CLNs [24], [29], which may be associated with immune surveillance of the CNS. In addition, myelin antigens presented by DCs have been detected in the CLNs of a primate model of an inflammatory demyelinating disorder [29].

**Figure 3:**
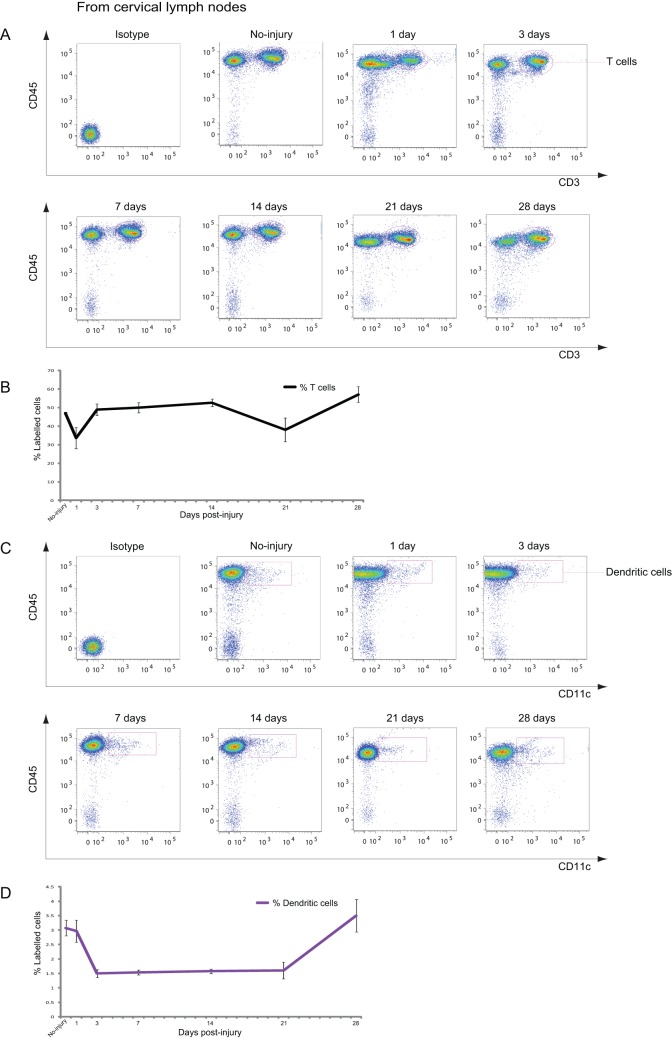
T cells and DCs in the cervical lymph nodes (**CLNs**) **after CCI.** A: Representative cytometry data for T cells in the CLNs at the indicated days after CCI. B: Graph illustrating quantitative data for T cells in the CLNs after CCI. C: Representative cytometry data for DCs in the CLNs at the indicated days after CCI. D: Graph illustrating quantitative data for DCs in the CLNs after CCI. (B, D) *n* = 3–4 for each experiment. T cells: * *p*<0.05 at 28 dpi compared with 1 dpi; DCs: ** *p*<0.01 at 3 dpi compared with no injury, * *p*<0.05 at 7, 14, and 21 dpi compared with no injury.

**Figure 4:**
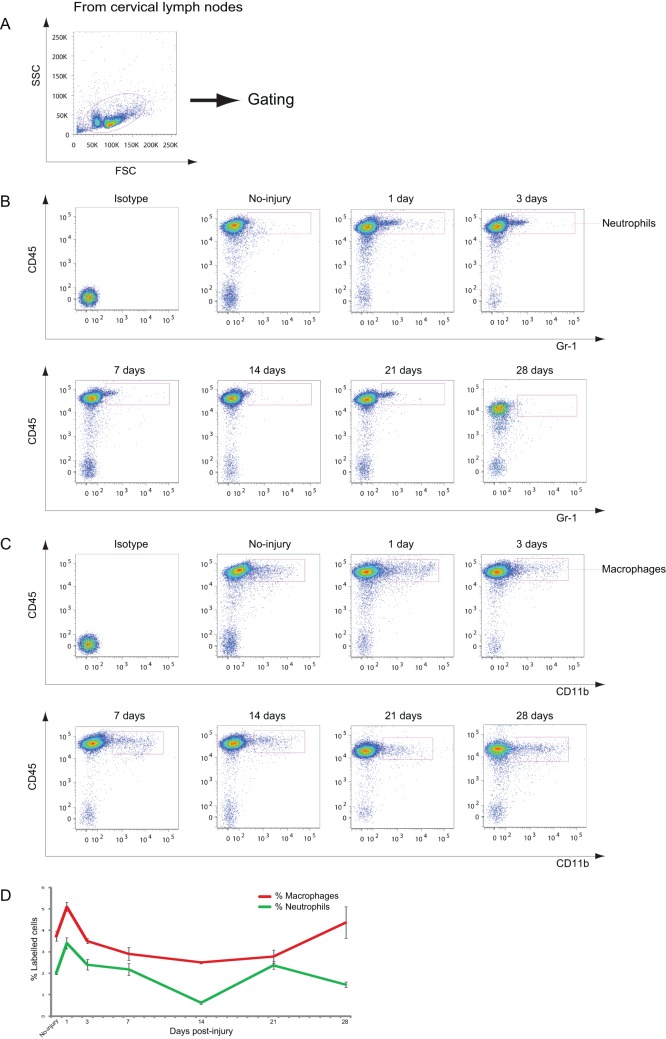
Neutrophils and macrophages in the CLNs after CCI. A: Dot plots of immune cells in the CLNs for live cell analysis. B: Representative cytometry data for neutrophils in the CLNs at the indicated days after CCI. C: Representative cytometry data for CD45^+^/CD11b^+^ macrophages in the CLNs at the indicated days after CCI. D: Graph illustrating quantitative data for neutrophils and macrophages in the CLNs after CCI. *n* = 3–4 for each experiment. Neutrophils: ** *p*<0.01 at 1 and 14 dpi compared with no injury; macrophages: ** *p*<0.01 at 7, 14, and 21 dpi compared with 1 dpi.

However, only fragmentary information is available on the dynamics of immune reactions in the injured CNS, as well as in the periphery. In the present study, the accumulation of neutrophils, macrophages, T cells, DCs, and microglia was quantified by flow cytometry in the injured brain, the CLNs, and spleen up to 4 weeks after controlled cortical impact (CCI), an experimental model of TBI. Immunohistochemical analysis was also performed to characterize the microglia and macrophages accumulated in the brain.

**Figure 5:**
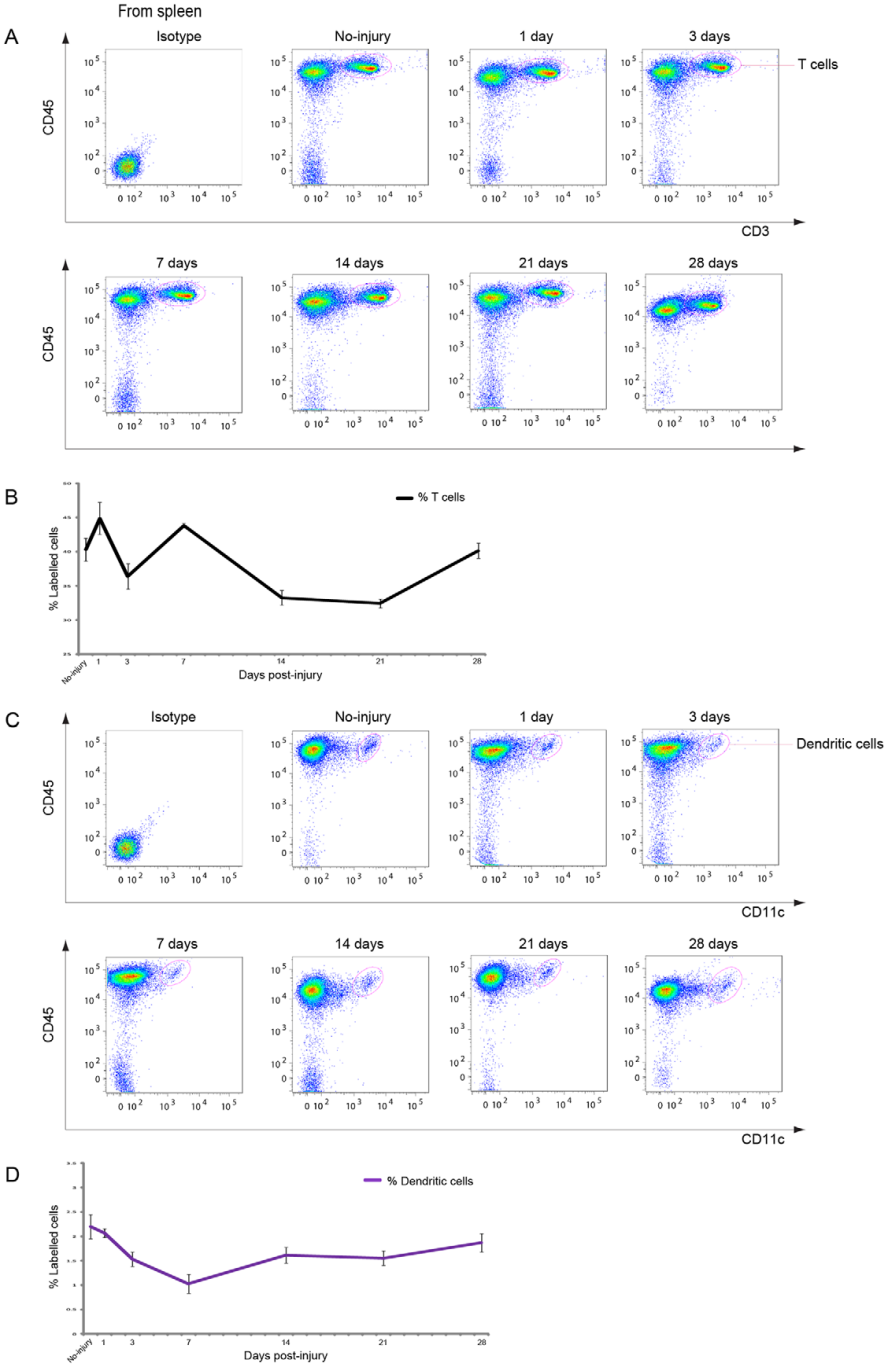
T cells and DCs in the spleen after CCI. A: Representative cytometry data for CD45^+^/CD3^+^ T cells in the spleen at the indicated days after CCI. B: Graph illustrating quantitative data for T cells in the spleen after CCI. C: Representative cytometry data for DCs in the spleen at the indicated days after CCI. D: Graph illustrating quantitative data for DCs in the spleen after CCI. (B, D) *n* = 3–7 for each experiment. T cells: * *p*<0.05 at 14 and 21 dpi compared with no injury, * *p*<0.05 at 3 dpi compared with 1 dpi, ** *p*<0.01 at 14 and 21 dpi compared with 1 dpi; DCs: ** *p*<0.01 at 7 dpi compared with no injury, * *p*<0.05 at 28 dpi compared with 7 dpi.

**Figure 6:**
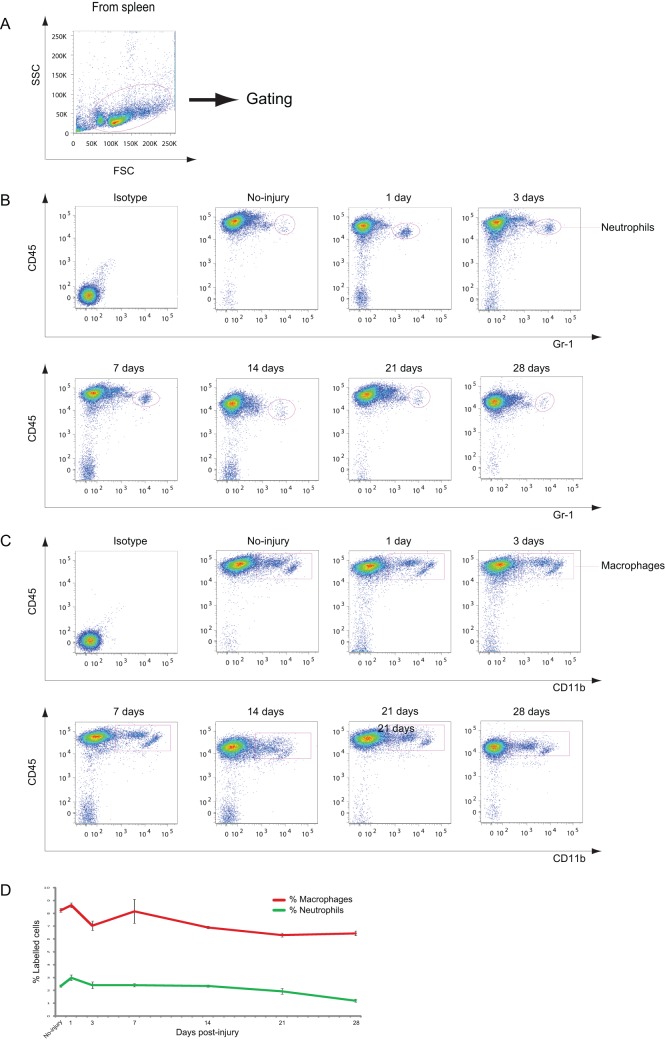
Neutrophils and macrophages in the spleen after CCI. A: Dot plots of immune cells in the spleen for live cell analysis. B: Representative cytometry data for neutrophils in the spleen at the indicated days after CCI. C: Representative cytometry data for macrophages in the spleen at the indicated days after CCI. D: Graph illustrating quantitative data for neutrophils and macrophages in the CLNs after CCI. *n* = 3–5 for each experiment. Neutrophils: ** *p*<0.01 at 28 dpi compared with no injury.

**Figure 7:**
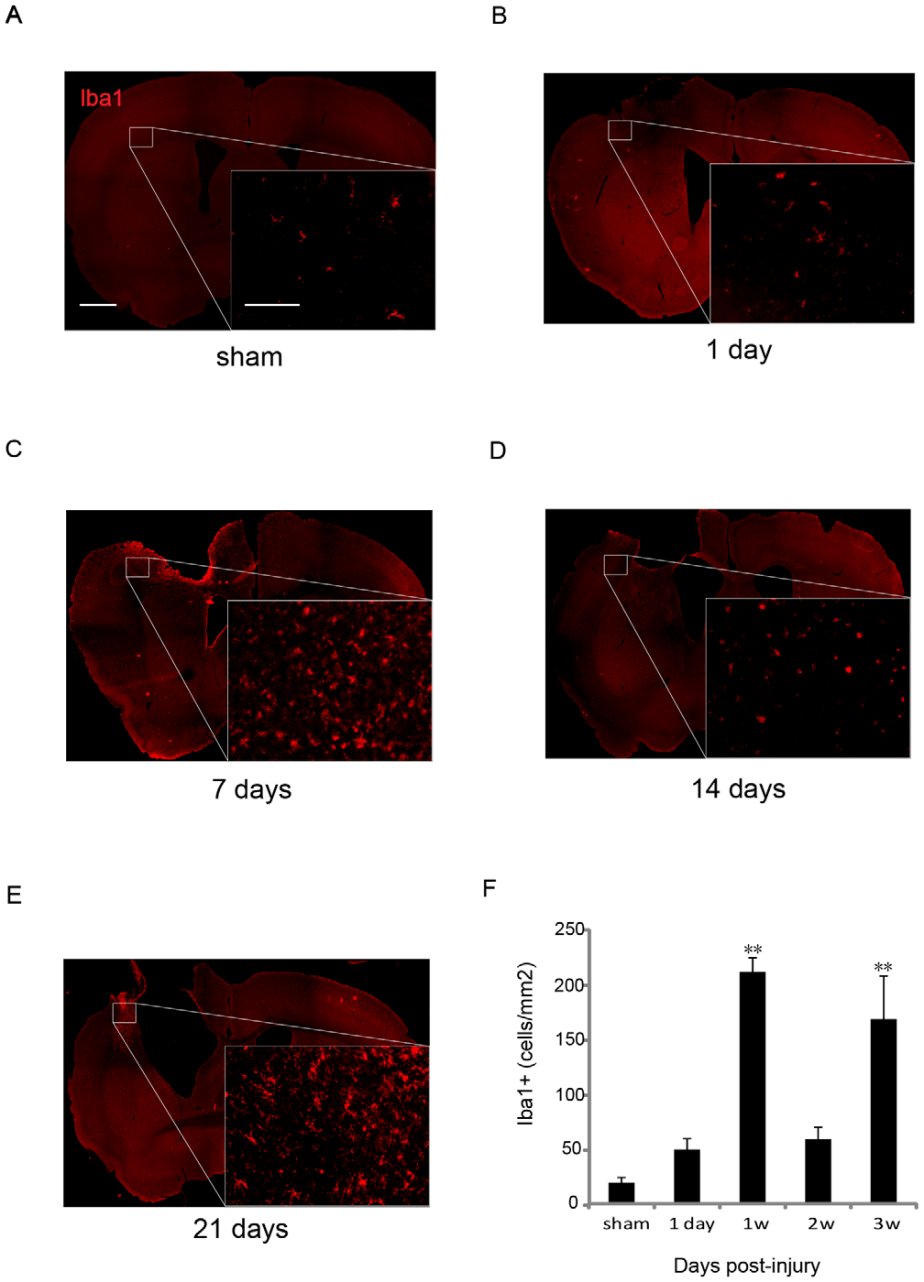
Bimodal increase in the Iba-1-positive cells in the brain after CCI. (A–E) Brain sections immunostained for Iba-1 at the indicated days after CCI. The red signals show Iba-1-positive microglia/macrophages in sham-operated mouse brains (A) and in brains after CCI at 1 dpi (B), 7 dpi (C), 14 dpi (D), and 21 dpi (E). The small image on the right lower side of each large image is a magnified view of the square indicated. Scale bars: large image, 600 µm; small image 100 µm. (F) The graph shows the number of microglia/macrophages at the indicated days after CCI. Microglia/macrophages observed around the injured side significantly increased at 7 dpi and 21 dpi. Sham, sham-operated mice. *n* = 4 for each group. ** *p*<0.01 at 7 and 21 dpi compared with sham controls.

**Figure 8:**
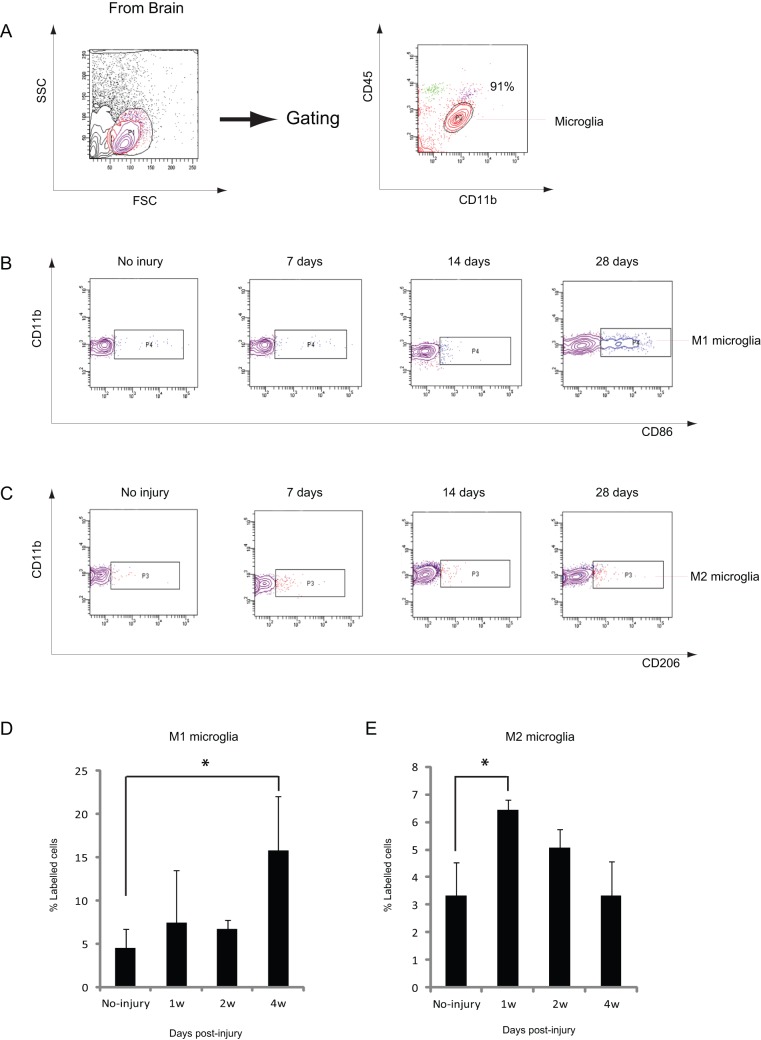
Changes in specific subsets of microglia in the injured brain. A: Representative cytometry data showing the purity of microglia isolated. Of the isolated cells, 91% were microglia. B: Representative cytometry data for CD86^+^/CD11b^+^ M1-like microglia in the brain at the indicated days after CCI. C: Representative cytometry data for CD206^+^/CD11b^+^ M2-like microglia in the brain at the indicated days after CCI. D: Graph illustrating quantitative data for CD86^+^/CD11b^+^ cells in the brain after CCI. CD86^+^/CD11b^+^ cells were higher in number than in the intact brain at 28 dpi. E: Graph illustrating quantitative data for CD206^+^/CD11b^+^ cells in the brain after CCI. CD206^+^/CD11b^+^ cells were higher in number at 7 dpi than in the intact brain, and decreased thereafter until 28 dpi. *n* = 3–5 for each group. * *p*<0.05 compared with no injury.

## Materials and Methods

### Mice

C57B6/J mice were purchased from Japan SLC Inc. (Shizuoka, Japan) and were housed in specific pathogen-free conditions. All the mice were treated and cared for in accordance with the guidelines of Osaka University that pertained to the treatment of experimental animals.

### CCI

Adult male C57B6/J mice (8–10 weeks old) were anesthetized with sodium pentobarbital (60 mg/kg intraperitoneal injection; Kyoritsu Seiyaku, Tokyo, Japan) and were stabilized using a stereotaxic frame (Muromachi, Tokyo, Japan). The scalp was retracted and circular craniotomy (diameter, 4 mm) was performed over the left sensorimotor cortex. The craniotomy was performed with a drill, with the center located at 0 mm anteroposterior to and 2 mm lateral to the bregma. Cortical traumatic injury was induced using a pneumatic impact device (Amscien Instruments, Richmond, VA USA), as previously reported [30]. Once the dural surface was exposed, the position of the tip of the impactor (diameter, 3 mm) was adjusted to the center of the opened surface. The impact was made at a depth of 1.0 mm, a speed of 4.0–4.5 m/s, and a duration of impact of 120 ms. After the impact, the removed skull was returned to its original position, the scalp was sutured, and the mice were allowed to awaken. Mice who underwent sham operations served as controls. In these mice, all surgical procedures were performed, with the exception of cortical traumatic injury.

### Preparation of Leukocytes

Leukocytes were prepared as previously reported [31], [32], with minor modifications. At 1, 3, 7, 14, 21, and 28 d post-injury (dpi), the mice were transcardially perfused with ice-cold phosphate buffered saline (PBS), and the brain, CLNs, and spleen were rapidly removed. The injured left hemispheres and CLNs were placed separately into 10 mL of ice-cold Hanks' balanced salt solution (HBSS; Life Technologies, Carlsbad, CA) containing 3% fetal bovine serum (FBS; MP Biomedicals, Irvine, CA). The dissociated tissues were suspended and digested with collagenase D (2.5 mg/mL; BD Biosciences, Franklin Lakes, NJ) and DNase I (0.25 mg/mL; Sigma-Aldrich, Saint Louis, MO) at 37°C in a water bath shaker for 45 min. The resultant tissue homogenates were mechanically dissociated and were passed through a 70-μm nylon cell strainer (BD Biosciences). The cell suspension was centrifuged at 700 *g* for 5 min at room temperature. Subsequently, the CLN pellet was washed in flow cytometry staining buffer (BD Biosciences) for cell surface staining, while the brain pellet was resuspended in 5 mL of 30% Percoll (GE Healthcare, Little Chalfont, UK) diluted in HBSS medium and overlaid on 3.5 mL of 70% Percoll solution. The gradient was centrifuged at 1000 *g* for 25 min at room temperature. Finally, leukocytes collected from the brains were gently removed from the interface and washed using flow cytometry staining buffer for cell surface staining.

Spleens were removed from the mice, pooled into ice-cold PBS, and mechanically disrupted. They were then washed twice, resuspended in HBSS medium, and centrifuged at 350 *g* for 5 min at room temperature. Red blood cells from each spleen were then lysed in 5 mL of 1× RBC lysis buffer (BioLegend, San Diego, CA) for 5 min on ice, followed by dilution of the lysed sample with 20 mL of PBS. The cells were spun down by centrifugation at 350 *g* for 5 min at room temperature. The cells were washed twice with the flow cytometry staining buffer for cell surface staining.

### Preparation of Microglia

Microglia were isolated using an optimized method, as described by de Hass [33]. The mice were transcardially perfused with ice-cold PBS until colorless fluid appeared from the inferior vena cava. The injured hemispheres were immediately dissected and placed in Hank's balanced salt solution (HBSS, Life Technologies 14170–088), containing 15 mM HEPES (Life Technologies 15630–056) and 0.5% glucose (Sigma G8769). The brains were gently dissected into small pieces and were centrifuged at 1000 *g* for 10 min at 4°C. The pellets were then suspended in HBSS-containing 0.25% trypsin (Life Technologies 15090–046) and incubated for 15 min in a 37°C water bath with intermittent shaking. Trypsin activity was inhibited by the addition of an equal volume of ice-cold HBSS containing 20% heat-inactivated fetal bovine serum (FBS, Life Technologies 10108–165) and 100 µg/mL trypsin inhibitor (Sigma T6522). The cells were pelleted at 1,000 *g* for 10 min at 4°C and resuspended in 5 mL ice-cold HBSS, followed by titration using Pasteur pipettes of decreasing diameter (VWR International 612–1701), and the cell suspension was filtered through a 70-µm cell strainer (BD Biosciences 352350) and rinsed in ice-cold HBSS. Finally, cells were pelleted at 1,000 *g* for 10 min at 4°C. To yield a stock isotonic Percoll solution, 9 volumes of Percoll (GE Healthcare 17–0891) were mixed with 1 volume of 10× PBS. Different Percoll densities were obtained via dilution with 1× PBS. Cell pellets were resuspended in 3.3 mL of ice-cold 75% Percoll and were gently overlayered with 5 mL of ice-cold 25% Percoll and subsequently with 3 mL of ice-cold PBS. The density gradient was centrifuged at 800 *g* for 25 min at 4°C. After centrifugation, a thick myelin-containing layer at the 0/25 interface was removed with a Pasteur pipette and the cells at the 25/75 interface were collected with a fresh Pasteur pipette. To pellet these cells, the cell-Percoll suspension was diluted at least threefold with ice-cold PBS and centrifuged at 1,000 *g* for 10 min at 4°C. Finally, the cells were washed twice with 2 mL of flow cytometry staining buffer (BD Biosciences) for cell surface staining and flow cytometry analysis.

### Cell Surface Staining and Flow Cytometry Analysis

For cell surface staining, anti-mouse CD45-PE/Cy7, anti-mouse CD11b-PE, anti-mouse granulocyte-differentiation antigen-1 (Gr-1)-FITC, anti-mouse CD11c-Pacific Blue™, and anti-mouse CD3-APC (BioLegend) were used. Anti-mouse CD45-PE/Cy7, anti-mouse CD11b-PE, anti-mouse CD86-APC, and anti-mouse CD206-FITC were also used for detection of M1-like and M2-like microglia. The cells were suspended in flow cytometry staining buffer (BD Biosciences) and treated with Fc-receptor blocker (anti-mouse cluster of differentiation 16/32 (CD16/32) antibody (BioLegend) for 20 min at 4°C in order to eliminate nonspecific binding of antibodies to Fc receptors. Next, the cells were stained by these antibodies for 30 min at 4°C at dark. The samples were washed twice in 2 mL of flow cytometry staining buffer and resuspended in 500 μL of the staining buffer per sample.

Flow cytometry was performed with a BD FACSCanto II (BD Bioscience) and analyzed using BD FACSDiva software (BD Bioscience) and the FlowJo software (version 9.3.1; TreeStar, Inc., Ashland, OR). The specificity of the signals of antibodies against specific antigens was determined by performing control experiments using isotype-matched immunoglobulins (BioLegend).

### Immunohistochemical Analysis

The mice were transcardially perfused with 4% paraformaldehyde. Their brains were dissected and post-fixed in the same fixatives overnight at 4°C. On the following day, the brains were immersed in 30% sucrose in PBS at 4°C for another night; subsequently, they were embedded in optimal cutting temperature (OCT) compound and were stocked at −80°C immediately after freezing on dry ice. A series of 20-µm coronal sections were cut on a cryostat and mounted on MAS-coated slides (Matsunami Glass, Osaka, Japan). The sections were washed 3 times for 5 min in PBS and were blocked with blocking buffer containing 5% bovine serum albumin/0.1% Triton X-100/PBS for 1 h at room temperature. Rabbit anti-Iba1 (1∶250, Wako, Japan) was used as the primary antibody and was diluted in blocking buffer. After incubation with the primary antibody overnight at 4°C, the sections were washed 3 times for 10 min with PBS-T containing 0.05% Tween 20 and PBS and incubated with secondary antibody diluted in PBS-T (Alex-568-conjugated goat anti-rabbit IgG [1∶500, Invitrogen]) for 1 h at room temperature. Sections were observed with a fluorescence microscope (BZ-9000, Keyence, Osaka, Japan). Image processing analysis and measurements were performed using Image J software (National Institute of Health, USA). The number of Iba-1^+^ microglia was counted in every 100 µm section within the lesion site and cumulative number was calculated.

### Statistics

All the values are expressed in terms of mean ± standard error of mean. Quantitative data were analyzed using the Tukey-Kramer method between 2 groups for all the combinations used in each experiment. Values of *p*<0.05 were considered statistically significant.

## Results

### Time Course of the Changes in Neutrophil, Macrophage, and Microglial Accumulation in the Injured Brain after CCI

The time course of the accumulation of immune cells in the injured brain was analyzed using isolated cells from the injured side of the cerebral cortex that were gated for live cell analysis (Figure 1A). Neutrophils positive for CD45 and Gr-1 accumulated in the injured brain, increasing to approximately 12% of the gated cells at 1 dpi and then decreased by 7 dpi (Figure 1B). CD45 and CD11b were used as markers for identification of macrophages and microglia. Two distinct populations of macrophages and microglia were observed: CD45^high^/CD11b^+^ cells and CD45^low^/CD11b^+^ cells. CD45^high^/CD11b^+^ cells were considered macrophages and CD45^low^/CD11b^+^ cells were considered microglia [34], [35]. A transient increase in the number of macrophages was observed at 1 and 3 dpi (Figure 1C, D) and the number of macrophages decreased thereafter. In contrast, the number of microglia gradually increased to approximately 10% of the gated cells at 7 dpi (Figure 1C, D), followed by a decrease to below the baseline level at 2 weeks (14 dpi), and then another increase at 3 and 4 weeks (21 and 28 dpi, respectively). Therefore, microglia show a bimodal increase in the injured brain after CCI.

### T Cells and DCs Infiltrate the Brain after CCI

The accumulation of DCs and T cells in the CNS was examined after CCI. CD45^+^/CD11c^+^ cells and CD45^+^/CD3^+^ cells were determined to be DCs and T cells, respectively (Figure 2A–C). Both types of the cells infiltrated the injured brain, and the numbers of both cell types peaked at 3 dpi (Figure 2D). However, the increase in these cells only reached approximately 1% of the gated cells, and the percentage of both DCs and T cells was much less than that of neutrophils, macrophages, and microglia (Figure 1). From 1 to 4 weeks (7–28 dpi), DCs gradually decreased and returned to the baseline level (Figure 2D). Although the number of T cells also decreased at 1 and 2 weeks (7 and 14 dpi, respectively), the number of T cells again rose slightly but significantly at 28 dpi (Figure 2D). Throughout the study period, more T cells than DCs were present in the brain (Figure 2D).

### Dynamic Changes in Immune Cells in the CLNs after CCI

The CLNs are considered the draining lymphatic organs of the brain [24], but the roles of these organs remain elusive when the brain is under pathophysiologic conditions. Therefore, the dynamics of leucocytes in the CLNs after CCI were examined. The number of CD45^+^/CD3^+^ T cells reduced from 46.9% to 33.7% at 1 dpi; returned to the baseline level at 3, 7, and 14 dpi (Figure 3A, B); followed by a slight decrease in number at 21 dpi; and finally, an increase to over the baseline level at 28 dpi. The number of DCs in the CLNs reduced from 3.1% to 1.5% at 3, 7, 14, and 21 dpi, and then increased to 3.5% at 28 dpi (Figure 3C, D). Neutrophils and macrophages increased transiently at 1 dpi and returned to the baseline level at 3 and 7 dpi (Figure 4A-D), respectively. Neutrophils decreased to less than 1% at 14 dpi and returned to the baseline level at 21 and 28 dpi (Figure 4D).

### Alterations in T cells in the Spleen after CCI

The number of leucocytes in the spleen after CCI was examined in order to understand the systemic state of immune cells after brain injury. T cells are the dominant cell type in the spleen. The number of T cells decreased from 1 to 3 dpi (Figure 5A, B), followed by a further decrease to approximately 30% at 14 and 21 dpi. This dynamic pattern of changes in the numbers of T cells was similar to that found for T cells in CLNs (Figure 5A, B).

### Profile of DCs, Neutrophils, and Macrophages in the Spleen after CCI

The DCs in the spleen decreased from 2.2% to 1.0% at 7 dpi, and then gradually increased to approximately 1.6% at 14–28 dpi (Figure 5C, D). The number of neutrophils in the spleen did not change significantly (approximately 1–2%) during the observation period (Figure 6A, B). The macrophages in the spleen also did not change significantly during this period (Figure 6C, D).

### Immunohistochemical Analysis of Microglia and Macrophages in the Brain after CCI

As interesting bimodal changes were observed in the number of microglia in the injured brain after CCI, immunohistochemical analysis for Iba1 was performed to further examine the changes in microglia and macrophages in the brain after CCI (Figure 7). Intense signals for Iba-1 were observed around the site of injury at 7 and 21 dpi after CCI (Figure 7 C, E). The number of Iba1-positive microglia/macrophages around the injured site at 7 dpi was higher than that in sham-operated mice (Figure 7A, C, F). Iba-1 positivity decreased at 14 dpi (Figure 7D, F) and increased again at 21 dpi (Figure 7E, F). These results are consistent with those obtained by flow cytometry. Morphologic analysis showed that Iba1-positive microglia/macrophages were round at 7 dpi (Figure 7C) but had a predominately ramified shape at 21 dpi (Figure 7E). These results suggest that different subsets of microglia play distinct roles at 7 and 21 dpi.

### Increase in Distinct Subsets of Microglia in the Injured Brain

The abovementioned observations prompted us to further characterize microglia in the brain. Macrophages have been classified into 2 subsets: pro-inflammatory M1 and anti-inflammatory M2. Both subsets are involved in the pathophysiology of inflammation in the CNS [36], [37]. As microglia are derived from monocytes during fetal life and have the same origin as macrophages, different subsets of microglia may contribute to pathophysiological developments in each phase after CCI. Therefore, the changes in specific subsets of microglia in the brain after CCI were analyzed. As CD86 is considered an M1 macrophage marker and CD206 an M2 macrophage marker [15], these markers were used to test alterations in 2 different subsets of microglia by flow cytometry. Of the CD45^low^/CD11b^+^ cells isolated from both the intact and injured brains, 91% were microglia (Figure 8A). Among them, CD86^+^/CD11b^+^ M1-like microglia significantly increased at 28 dpi but not at 7 or 14 dpi, after CCI (Figure 8B, D). CD206^+^/CD11b^+^ M2-like microglia at 7 dpi were significantly higher than those in intact controls, and their number gradually decreased thereafter (Figure 8C, E). These results suggest that different subsets of microglia increased in a bimodal manner after CCI.

## Discussion

Although previous studies measured mixed populations consisting of microglia and macrophages [15], [38], [39], this study assessed each of these 2 distinct populations separately, according to the intensity of CD45/CD11b immunofluorescence (Figure 1C). Neutrophils and macrophages strongly infiltrated the brain in the early phase of CCI, as would be expected on the basis of previous studies in a TBI model [40]–[42]. However, this study identified that microglia in the injured brain first increased and then reached a peak at 1 week after CCI, which was followed by a second surge after 2 weeks. A reduction in microglia in the injured brain was observed 1 d after CCI, followed by a bimodal increase at 1 week and in the chronic phase. This increase was predominantly found around the injury site (Figure 7). Iba1 staining of injured brain sections showed that microglia were morphologically round at 1 week after injury, whereas those at 3 weeks were more ramified, suggesting that different subtypes of microglia were dominant between 1 and 3 weeks after injury. Indeed, cell surface marker analysis showed that M2-like microglia peaked at 1 week and M1-like microglia increased at 4 weeks (Figure 8). However, more than 70% of microglia were CD86^−^/CD206^−^. Studying other markers for M1 and M2 might be necessary to appropriately classify microglia in the brain, or unknown subsets of microglia might differentiate from resting state microglia after CCI. Complete characterization of microglia will be required to elucidate the function of these cells.

Interestingly, the dynamic changes seen in the number of T cells in the CLNs showed a similar pattern, with a 1-week delay, to that of microglia in the injured brain. Thus, it is possible that T cells in the CLNs respond to activated microglia in the injured brain. Several studies suggest that CSF flows into CLNs in spite of the existence of the BBB [43]–[47]. Although microglia do not migrate to the CLNs (Figure 3D), microglia and T cells may come into contact with microglia in the meninges around the injured brain, as the meninges are the sites of interaction between the CNS and immune cells [48]. In the acute phase, T cells in the spleen also appear to respond to an increasing number of microglia in the injured brain after CCI (Figure 1D). The delay in the reaction of T cells is greater in the spleen than in CLNs, presumably because the spleen, as a draining lymphatic organ, is anatomically farther from the CNS than the CLNs are.

One day after CCI, neutrophils rapidly infiltrated the injured brain but returned to the baseline level at 7 dpi (Figure 1D). These data are consistent with previously reported findings in the TBI model [40]–[42]. However, the role of neutrophils in CNS injuries is controversial [41].

Accumulation of T cells and DCs was observed in the injured brain at 1 and 3 d after CCI, respectively. Since T cells accumulated at day 1, they are unlikely to have responded to antigen-specific acquisition, suggesting that early-accumulating T cells may be γδT cells, in particular IL-17-producing γδT cells that are pathogenic in ischemic brain injury [35].

Following this study, the next logical step is elucidation of the function of each type of immune cell in CNS injury. In particular, it would be important to elucidate the functions of microglia in the acute and chronic phases after CCI. This study can serve as the basis for future studies and offers new insights into the crosstalk between the CNS and the immune system in CNS injuries.
